# Defining the role and reach of a geriatrician

**DOI:** 10.1016/j.lanhl.2024.100644

**Published:** 2024-11

**Authors:** Matteo Cesari, Jotheeswaran Amuthavalli Thiyagarajan, Antonio Cherubini, Miguel Angel Acanfora, Prasert Assantachai, Mario Barbagallo, Mamadou Coume, Theresa Diaz, Nicholas Fuggle, Sonia Ouali Hammami, Kenneth Madden, Radmila Matijevic, Jean-Pierre Michel, Mirko Petrovic, Cornel Sieber, Nicola Veronese, Finbarr C Martin, Anshu Banerjee, John W Rowe

**Affiliations:** a Department of Maternal, Newborn, Child and Adolescent Health and Ageing, WHO, Geneva, Switzerland; b Geriatria, Accettazione Geriatrica e Centro di Ricerca per l'Invecchiamento, IRCCS INRCA, Ancona, Italy; c Department of Clinical and Molecular Sciences, Università Politecnica delle Marche, Ancona, Italy; d Instituto Ciencias de la Salud, Fundación Barceló, Buenos Aires, Argentina; e Faculty of Medicine Siriraj Hospital, Mahidol University, Bangkok, Thailand; f Geriatric Unit, Department of Medicine, University of Palermo, Palermo, Italy; g Faculté de Médecine, de Pharmacie et d’Odontologie, Université Cheikh Anta Diop, Dakar, Senegal; h MRC Lifecourse Epidemiology Centre, University of Southampton, Southampton, UK; i Faculty of Medicine, University of Monastir, Monastir, Tunisia; j Department of Medicine; University of British Columbia, Vancouver BC, Canada; k Faculty of Medicine; University of Novi Sad, Novi Sad, Serbia; l Department of Rehabilitation and Geriatrics, Geneva University Medical School, Geneva, Switzerland; m Section of Geriatrics, Department of Internal Medicine and Paediatrics, Ghent University, Ghent, Belgium; n Institute for Biomedicine of Aging, Nuremberg, Germany; o Population Health Sciences, King's College London, London, UK; p The Columbia Aging Center and the Department of Health Policy and Management, Mailman School of Public Health, Columbia University, New York, NY, USA

## Abstract

Population ageing is a global occurrence. Unfortunately, the shortage of health professionals with geriatric competencies is a major factor restricting high-quality care for older people worldwide. Strengthening the knowledge and skills of the health workforce to better respond to the needs of older people is a major global priority. Geriatricians can play a pivotal role in reorienting care for older people towards an integrated and person-centred care system focused on functional ability, preventive strategies, and age-friendly services. The current scenario requires efforts to be directed towards establishing a standardised competency-based definition of a geriatrician to allow for an accurate estimation of the existing workforce with adequate training in geriatrics as crucial resources to facilitate the paradigm shift. This Personal View, supported by the International Association of Gerontology and Geriatrics and the European Geriatric Medicine Society, proposes a standardised definition of a geriatrician based on expected competencies and roles and a precise description of the essential features of geriatric medicine. By reducing ambiguities and offering a competency-based framework, the current standardisation approach is expected to facilitate better support, monitoring, and allocation of resources for improving care for older people worldwide.

## Introduction

Population ageing is occurring at an unprecedented rate worldwide, and low-income and middle-income countries (LMICs) are experiencing the most substantial demographic shifts due to a combination of increased life expectancies and declining birth rates. Currently, over 1 billion older people (ie, people aged ≥60 years) reside in LMICs. By 2050, over 80% of older people are projected to reside in LMICs.^1^ As people age, the probability of experiencing age-related physiological changes and chronic diseases increases, resulting in physical and mental impairments.^2^ Moreover, the diverse range of health conditions and functional abilities among older people, known as clinical heterogeneity, affects their social roles and participation. To address the various changes in older people, it is important to challenge the traditional mono-dimensional, disease-driven approach.^3^

In view of the fact that “despite the predictability of population ageing and its accelerating pace, many health systems might not be sufficiently prepared to respond to the needs of the rapidly ageing population”, the UN General Assembly established the UN Decade of Healthy Ageing (2021–30) in 2020.^4^ The global initiative, coordinated by WHO, brings together governments, civil society, international agencies, professionals, academia, the media, and the private sector to improve the lives of older people and their families and communities. The vision of the UN Decade of Healthy Ageing (2021–30) is to create a world where everyone can live long, healthy lives and have the freedom to do and be what they value most. To realise the vision, four action areas and four enablers were identified (panel 1).^5^^,^^6^

Panel 1 **Action areas and enablers of the UN Decade of Healthy Ageing (2021–30)**

**Action areas**

1. Combating ageism: change how we think, feel, and act towards age and ageing
2. Age-friendly environments: ensure that communities foster the abilities of older people
3. Integrated care: deliver person-centred integrated care and primary health services that are responsive to older people
4. Long-term care: provide access to long-term care for older people who need care

**Enablers**

1. Listen to diverse voices and enable meaningful engagement of stakeholders, especially older people
2. Build capacity and nurture leadership to take appropriate action integrated across sectors
3. Connect stakeholders around the world to share and learn from the experiences of others
4. Strengthen data, research, and innovation to accelerate implementation

Of note, the vision of the UN Decade of Healthy Ageing (2021–30) is aligned with the agenda of Sustainable Development Goals, which recognises universal health coverage to be crucial to ensure healthy lives and promote wellbeing for all, at all ages by providing access to quality health services without financial hardship.^7^ Unfortunately, challenges such as ageism, pandemics, climate crises, wars, pollution, and systematic inequalities are hindering countries from achieving their Sustainable Development Goals. The challenges also negatively affect the countries’ ability to adapt to demographic changes and enhance the health and wellbeing of older people and their families.

A major factor limiting the availability of high-quality care for older people is the serious shortage of health professionals with geriatric competencies. The inadequacy of a care workforce devoted to older people is not restricted to LMICs but extends to wealthier countries as well.^8^ The current curricula for training health-care professionals still largely neglect the care needs specific to older people.^8^ Hence, strategies to improve the attitudes, knowledge, and skills of the health workforce to better respond to the needs and priorities of older people within a system of integrated, person-centred care are a major global priority.

## Role of geriatricians in the reorientation of care for healthy ageing

Geriatricians play an active leadership role in guiding the shift towards a person-centred and more integrated care system that focuses on functional ability, with emphasis on preventive strategies and age-attuned acute and rehabilitative services.^9^, ^10^, ^11^ The approach developed and used over the past decades in geriatric medicine is based on a comprehensive assessment of individuals and delivery of multidisciplinary, person-centred interventions that bridge clinical and social care.^9^ Moreover, geriatricians have continuously supported a capacity-oriented approach that considers the needs and priorities of older people, which includes promoting inclusiveness, adapting care practices, and addressing ageism.^12^

Because geriatricians are and will remain too few to address the needs of the increasing older population, especially in LMICs, it is essential to rethink how the expertise and experience of geriatricians, as well as their direct contributions, can be optimally used to enhance the effect of their expertise. For example, geriatricians should be more actively involved in multistakeholder collaborations to improve care for older people. Geriatricians should also disseminate the principles of geriatric care and prevention to the next generation of health-care workers,^8^^,^^13^^,^^14^ especially those involved at the primary health-care level.^15^^,^^16^ Changes required in clinical training and competencies need to extend beyond medical doctors and include all health-care providers who work with older people.

Quantification of the number of geriatricians worldwide is difficult at present because geriatricians are included by the International Labour Organization within the macro group of specialist medical practitioners (International Standard Classification of Occupations code 2212) together with all the other medical doctors who “diagnose, treat, and prevent illness, disease, injury, and other physical and mental impairments in humans using specialised testing, diagnostic, medical, surgical, physical, and psychiatric techniques through the application of the principles and procedures of modern medicine”.^17^ In other words, distinguishing specialists based on their competencies is not possible, which in turn hinders the estimation of available resources. At the same time, formal specialisation programmes in geriatrics are absent in many countries and are frequently insufficient in countries where specialisation programmes exist; furthermore, training curricula differ among countries.^18^^,^^19^

Thus, to have some uniformity across countries, it will be useful to standardise the definition of a geriatrician based on expected competencies and roles and then use the definition in national surveys to quantify the number of geriatricians. Furthermore, standardising the definition of a geriatrician will enable better support, monitoring, and allocation of resources for improving care for older people.

## Development of the definition of geriatrician

The International Association of Gerontology and Geriatrics (IAGG) is a global non-governmental organisation that represents gerontological organisations and focuses on promoting gerontological research and training. The mission of IAGG is to improve the quality of life and wellbeing of ageing individuals and society. The European Geriatric Medicine Society (EuGMS) is an umbrella organisation that promotes collaboration and coordination among national geriatric medicine societies in Europe. The main objective of EuGMS is to establish geriatric medicine as a recognised and independent medical specialty in all member states, contributing to improving the quality of care for all older people with age-related diseases. To the best of our knowledge, the EuGMS is, besides the IAGG, the only other association of national scientific societies on geriatrics.

In collaboration with the IAGG and EuGMS, WHO undertook an exercise to initiate the development of a competency-based definition for medical doctors specialised in the care of older people. The initiative is part of a broader activity aimed at defining all geriatric workforce cadres, including nurses, social workers, and others, to clearly define their roles and estimate their numbers and allow for better allocation of resources worldwide. The definition of a geriatrician was prioritised over that of other cadres as unequivocally related to the perimeter of action of geriatric medicine and the most recognised professional profile within the workforce of geriatrics. In other words, it was agreed that defining different health and care workers of a multidisciplinary geriatric team would be easier after the definitions of geriatric medicine and geriatricians are established.

As part of the collaboration, the IAGG gathered a group of recognised experts in geriatric medicine (n=14) from different regions. Then, the IAGG, in collaboration with WHO, developed and distributed a questionnaire to these experts to primarily capture, in a semistructured way, key concepts to be discussed and eventually represented in the final definitions (appendix p 1). Two online meetings were then organised with the experts to present and discuss the survey results. All the experts attended the meetings, and each of them had the opportunity to explain the rationale for their choices. The definitions provided by the working group were then evaluated, commented upon, and refined by representatives of the EuGMS.

## Geriatric medicine and geriatrician

The experts first agreed that a concise definition of a geriatrician required a clear description of the essential features of geriatric medicine, which is particularly important since other clinicians, focused on the care of older people, are already identified with specialty-specific titles (eg, geriatric psychiatrist, geriatric dentist, geriatric social worker, or geriatric nurse practitioner; panel 2).

Panel 2 **Key principles of geriatric medicine**

The practice of geriatric medicine is based on the comprehensive assessment and management, including health promotion and rehabilitation, of older people with declining or substantial loss of intrinsic capacity. By minimising losses of intrinsic capacities and mitigating their effects on functional abilities, geriatric medicine can improve health outcomes. The improved health outcomes include reduced morbidity and premature mortality, better experience of care, lower and more appropriate use of acute hospital facilities, and reduced dependence on institutional-based care.

The key principles of geriatric medicine include the following:

- focus on assessment, preservation, and optimisation of functional ability;
- life course perspectives that recognise the accrual of advantages and disadvantages over time based on lifestyle, exposures, environmental factors, and differential access to health care;
- management of older people with geriatric syndromes and complex clinical presentations (eg, frailty, multimorbidity, polypharmacy);
- promotion, facilitation, and support of interspecialty collaborations to improve care for older people, given the crosscutting nature of ageing;
- person-centred care based on a comprehensive assessment of functional, medical, and social factors to tailor interventions according to the priorities and needs of older people and achieve what matters the most to them;
- shared clinical decision making supported by an interdisciplinary team;
- goals of care that promote the comfort and dignity of older people;
- emphasis on clinical implications of the biology of ageing;
- care that spans the spectrum from population-based education and prevention to the management of acute illnesses and long-term care of chronic conditions; and
- provision of care and care integration in various settings, ranging from the community to acute and long-term care facilities.

After defining geriatric medicine, the collaborative process specifically aimed to define a specialist in geriatric medicine (panel 3). The experts recognised that many care providers who dedicate a substantial part of their clinical efforts to the care of older people might consider themselves to be geriatricians without the appropriate credentials or without having completed any special training, which are crucial issues, especially when formal accreditation is considered a prerequisite for recognition as a geriatrician and can even have implications for payment for services. At the same time, the demonstration that specific training has been completed remains a valuable formal way to document the achievement of a crucial set of competencies. To facilitate and disseminate the use of the definition, a simplified version is also provided (panel 3).

Panel 3 **Working definitions of a geriatrician**

**Simplified:** A geriatrician is a medical doctor who specialises in caring for older people and who formally possesses skills to assess and manage older people with medical and psychological issues, including social consequences.

**Detailed:** A geriatrician is a medical doctor who specialises in caring for older people. Geriatricians are usually board certified either in geriatric medicine (in countries where geriatric medicine is a recognised medical specialty) or, more often, in internal medicine or family medicine, with subsequent advanced clinical training and certification in geriatric medicine. The training and certification processes differ depending on the country and could affect the scope of practice.

Geriatricians possess specialised skills to assess and manage older people with medical and psychological issues, as well as their social consequences; these issues are characterised by complex clinical presentations and include geriatric syndromes such as frailty, mobility disorders, falls, incontinence, malnutrition, pressure ulcers, dementia, and delirium. A geriatrician’s skills are fundamental to delivering comprehensive, high-quality care to older people during acute episodes, when managing long-term conditions, and when providing rehabilitation.

Geriatricians are experts in promoting good health in later life, preventing illnesses, reducing disability, and judiciously using palliative care and end-of-life support by taking a capacity-oriented approach to address an older person’s needs and priorities.

Geriatricians respect the autonomy of older people and strive for well-informed, shared clinical decision making with patients. Geriatricians also discuss the plan for transferring care across settings (eg, hospital discharge arrangements) with older people and their care givers and involve other care partners to ensure the continuum of care. Geriatricians are usually involved in leading and guiding a multidisciplinary team of health-care professionals and understand and respect the expertise and skills of the other team members.

No specific chronological age qualifies older people for geriatric care because advancing age is characterised by substantial heterogeneity between individual trajectories and the experience of ageing-related declines in health.

A crucial issue discussed during the development of the definition of geriatric medicine was the opportunity to set a specific chronological age of the older person as an eligibility criterion. Although the skills of geriatricians are to be used with older people, setting a chronological age for general application is unhelpful for the following reasons. First, in any adult population and for any age group, the older the group, the more likely the individuals in the group will be living with multimorbidity or frailty, or both. In addition, any age group is characterised by marked heterogeneity. The prevalence and incidence of the conditions vary internationally across age, sex, and socioeconomic groups (as does life expectancy). Second, although focusing on older people most likely to benefit from the geriatrics approach is advocated in all health-care systems, the uncertainty with respect to the availability of a suitable health-care workforce suggests that some degree of local prioritisation is needed so that access can be more inclusive as resources develop.

A strict age-based criterion for access (eg, 65 years and older) is used in some systems and has the merits of clarity and administrative simplicity; however, such an arrangement will miss some older people who could benefit and include others for whom the value addition is less. Conversely, regarding clinical services as geriatric services or responsible medical doctors as geriatricians on the basis of caseload of patients with age or complexity alone would be misleading when the criteria of specialty services or have specialist competencies are not fulfilled.

Only formal training can ensure an adequate understanding of the ageing process, appropriate assessments, and proper decisions when dealing with the clinical complexity of geriatric patients.^14^^,^^20^ Preliminary evidence suggests that a geriatrician’s care can manage complex older patients more efficiently, thereby potentially improving the sustainability of health-care systems.^21^ Many medical doctors attend to older people with geriatric syndromes (ie, clinical conditions of older people that do not fit into specific disease categories^22^) but that does not make them geriatricians.^23^^,^^24^

## Discussion

Previous studies have described the profile of a geriatrician (even with suitable training curricula).^25^, ^26^, ^27^, ^28^ Some of these studies have also indicated the core activities that should characterise the discipline.^28^ The definitions proposed in this Personal View do present some overlap with the definitions mentioned in the previous studies, as our group of experts based their statements on existing evidence and the traditional foundations of geriatrics. However, to the best of our knowledge, this Personal View represents the first attempt to provide a definition that has been elaborated from the collaborative action of an international group of experts drawn from high-income countries and LMICs. Of note, the effort represents the complementary strengths of the IAGG and EuGMS, combining IAGG’s global reach with EuGMS’s unique capacity to specifically represent geriatricians across multiple countries.

As part of the UN Decade of Healthy Ageing (2021–30), countries have committed to reorient their care systems to focus on delivering person-centred care to older people, which includes capacity building of the health-care workforce.^6^ However, challenges exist due to insufficient standard definitions and data for several cadres that are part of the geriatric workforce. Strengthening competencies in geriatric medicine across a wide range of care settings, from hospitals to long-term care and community facilities, is crucial. Such strengthening of competencies requires measuring the current and future demands for improving care for healthy ageing and monitoring the progress towards Sustainable Development Goals. However, to accurately estimate the available geriatric workforce, a standard definition is mandatory. The current process of classifying occupations by the International Labour Organization does not adequately distinguish them according to competencies, thereby not facilitating clear measurement and monitoring of the specific needs of a country in the field of geriatric medicine. Therefore, for advancements in geriatric medicine and estimation of the number of geriatricians, a logic model has been established, based on the definition of a geriatrician’s functions and services, to identify the required competencies.

Several studies have reported extreme heterogeneity in the recognition and positioning of geriatric medicine, the role of geriatricians, and their training curriculum across countries.^18^^,^^29^^,^^30^ In settings that formally recognise geriatric medicine, the discipline might exist as a medical specialty or a subspecialty of other disciplines. Another important heterogeneity is also encountered in terms of the role of geriatricians across countries. For example, the primary place of geriatric care in most European countries is the hospital acute care unit,^18^^,^^31^ which is not the same in other countries where geriatricians might more likely be involved in community medicine and long-term care (eg, Asia) or in care systems relying on hospitalists (eg, USA).^31^ Moreover, the duration of geriatric training can vary substantially from country to country, ranging from 1 to 9 years.^29^^,^^30^

Although extreme variety also exists in the ratio between the number of geriatricians and the size of the older population, the shortage of geriatricians seems to be quite common.^18^^,^^31^^,^^32^ The issue of the shortage of geriatricians, also due to the still poor visibility and recognition of geriatric medicine,^32^ is particularly burdening for LMICs that are experiencing exceptionally rapid ageing of their populations at present. Given the urgent need for action, solely training geriatricians might not be the solution. Developing parallel preventive strategies that can facilitate access to the continuum of integrated, person-centred care through the strengthening of primary health care has now become necessary.

Geriatricians, even if few in number, can guide the change of care paradigms, promoting population-level initiatives for healthy ageing, leading reorientation of care towards the priorities and needs of older people, and supporting capacity building of health and care workers.^33^ Furthermore, several countries are exploring the possibility of improving the integration of geriatric education into existing residency programmes (eg, internal medicine^34^ and family medicine^35^) or into the undergraduate curriculum.^36^ In other words, geriatricians can play a crucial role in bringing geriatric care to settings and services in which older people are most frequently managed. This step will increase the quality of the offered care and generate a filter (preferably at the community level) to only evaluate and manage the most complex and frailest cases. The proposed strategy is consistent with the strengthening of the primary health care advocated by WHO, ensuring the sustainability of the system and the reorientation of care towards more preventive, person-centred, and integrated models.^3^

The present study has some limitations. The group of experts might not be representative of geriatricians worldwide, especially in terms of geographical and sex distribution. In this context, a potential argument is that the involvement of the EuGMS might have driven the working definition predominantly towards European standards. Although the argument could constitute a possible bias, the EuGMS is a unique organisation that represents many national geriatrics societies, with no equivalent worldwide. The absence of similar associations in other continents would have forced us to individually refer to the many national societies across countries, challenging the feasibility of our initiative.

Similarly, although the adopted method attempted to embrace a global approach to the theme, the definition of a geriatrician proposed in this Personal View was unavoidably influenced by the predominant presence of geriatric medicine in high-resource settings where the field is best established. A specific effort was made to include substantial input from experts in LMICs. Of note, the working definition provided in the current study should be further tested and refined (eg, through specific surveys) to capture the possible heterogeneities present at the global level.

Another limitation is the exclusive involvement of experts in geriatric medicine, and not other care workers, in the development of the working definition. However, the adopted approach is motivated by the need to clarify the frequently misunderstood work and responsibilities of a geriatrician, again due to the scarce training of the care workforce in geriatric medicine.^37^

Based on the limitation, a geriatrician’s profile might evolve in the future, especially considering the dynamic scenario brought about by the ageing population. In fact, the standardisation of the definition could allow an improved positioning of geriatricians in the health-care system and give more value to specific competencies.

The definitions proposed in this Personal View represent the first step towards standardising geriatric medicine and geriatricians. The definitions could serve as templates that need to be tested in the field for eventual adaptation and improvement of their global representativeness.

To estimate the geriatric workforce and needs, definitions based on the competency framework need to be developed for all cadres of the geriatric workforce, in collaboration with representative societies and associations. The definitions can then be applied and pilot tested for their feasibility by collecting data across countries. Thus, establishment of a data-collection mechanism in consultation with the IAGG, EuGMS, and other geriatric associations has been planned at the regional and national levels, which will help to establish a need-based forecast model for the geriatric workforce.

## Conclusions

Global population ageing reflects major scientific and public health advancements. Despite being a great accomplishment and a source of many opportunities, population ageing also presents substantial challenges, especially for health-care and social care systems and economies. The paucity of sufficient expertise in the care of older people in the general category of clinicians is aggravated by a severe and worsening shortage of geriatricians, in addition to ageist attitudes towards older people. Clarifying the characteristics, competencies, and role of the health and care workers belonging to the geriatric workforce, starting in this case from the geriatrician, is a crucial step towards more resilient systems and the delivery of more appropriate care aimed at the unmet needs of older people.

## Declaration of interests

JWR received a research grant from the Commonwealth Fund and has leadership roles in Red Cell Partners, Research Network on an Aging Society, and the Executive Committee of the Urban Institute. All other authors declare no competing interests.
