# Supplementary material for: Defining the role and reach of a geriatrician
Source: Lancet Healthy Longev. 2024 Nov;5(11):None. doi: 10.1016/j.lanhl.2024.100644 (PMC11602445; doi:10.1016/j.lanhl.2024.100644)
Supplement: Supplementary appendix [file mmc1.pdf]

THE LANCET
Healthy Longevity

Supplementary appendix

This appendix formed part of the original submission and has been peer reviewed.
We post it as supplied by the authors.

Supplement to: Cesari M, Amuthavalli Thiyagarajan J, Cherubini A, et al. Defining the role and reach of a geriatrician. *Lancet Healthy Longev* 2024. https://doi.org/10.1016/j.lanhl.2024.100644

Appendix 1. Questions included in the survey conducted in the IAGG working group to identify the main characteristics of a geriatrician.

1. Should only physicians be considered 'geriatricians', or should the term also apply to other health care providers such as nurses, social workers, etc.?
2. Should only internists and family practitioners be eligible for consideration as 'geriatricians', or can the term be applied to other specialties?
3. Should formal advanced training and certification be required to qualify as a geriatrician?
4. Should the definition of a geriatrician include a reference to the patient's age?
5. Should the definition of a geriatrician specify diseases treated?
6. Should the definition specify the type of provided care (e.g., acute vs chronic)?
7. Should the definition refer to settings of care?
